# Management of acute coronary syndrome in resource-limited set up: a summary of 4-year review of two hospitals in Ethiopia

**DOI:** 10.3389/fcvm.2025.1520899

**Published:** 2025-02-25

**Authors:** Dejene Atinafu Kebede, Tamirat Godebo Woyimo, Megersa Negesa Geleta, Seifu Bacha Chiri, Elsah Tegene Asefa, Kedir Negesso Tukeni

**Affiliations:** ^1^ Division of Cardiology, Department of Internal Medicine, Haramaya University, Harar, Ethiopia; ^2^ Division of Cardiology, Department of Internal Medicine, Jimma University, Jimma, Ethiopia; ^3^ Division of Cardiology, Department of Internal Medicine, Ambo University, Ambo, Ethiopia; ^4^ Division of Cardiology, Department of Internal Medicine, St. Paul Millennium Medical College, Addis Ababa, Ethiopia

**Keywords:** acute coronary syndrome, STEMI, klipp class, beta-blockers, PCI, heart failure, Ethiopia

## Abstract

**Introduction:**

Acute coronary syndrome refers to a group of diseases characterized by sudden, decreased blood supply to the heart muscle that results in cell death, also known as acute myocardial infarction. This results in severe chest pain or discomfort, with the subsequent release of cardiac biomarkers, and alterations in the electrocardiogram. It can cause diminished heart function and mortality if not treated properly with suitable measures. Despite the fact that percutaneous coronary intervention is the standard of care in one subset of acute coronary syndrome, significant number of patients were treated medically due to the limited service in the setting. The purpose of this study was to look at the patterns of acute coronary syndrome (ACS), as well as the management and outcomes of these patients in two Ethiopian tertiary institutions.

**Methods:**

A four-year retrospective study was undertaken on 308 patients with acute coronary syndrome, at two tertiary hospitals located in Ethiopia's capital-Addis Ababa.

**Results and Discussion:**

Of the 308 patients 72.4% were male, with the average age of 56.3 ± 13.5 years. Hypertension and diabetes were the two most common risk factors identified. The average time to present to the emergency room after symptom onset was 3.7 (SD ± 3.2) days. The majority of patients (67.9%) have been diagnosed with ST- Elevated Myocardial Infarction and were classified as Killip class I. Percutaneous Coronary Intervention was performed for 12.3% of patients, with the remaining receiving medical care. The average hospital stay was 8.51 (SD ± 7.2) days while In-hospital mortality was 8.8%. Tachycardia of >140 (AOR = 7.50, 95% CI: 1.36, 41.57), any degree of left ventricular dysfunction, Killip class IV (AOR = 6.03, 95% CI: 1.27, 28.61), and non-initiation of betablockers (AOR = 0.17,95% CI: 0.05, 0.63) were significantly associated with increased in-hospital mortality.

## Introduction

Acute coronary syndrome (ACS) refers to a group of cardiovascular diseases (acute myocardial ischemia, unstable angina, and/or myocardial infarction) characterized by an abrupt drop in coronary blood flow (1). The World Health Organization (WHO) predicts that cardiovascular disease (CVD) will remain the top cause of death globally until 2030 (2). Acute coronary syndrome (ACS), which in most of the case a complication of ischemic heart disease (IHD), is a prominent cause of mortality and morbidity, accounting for half of all CVD fatalities and over 2.5 million hospitalizations worldwide each year (3). IHD is the second most cause of death for both men and women in Europe, accounting for 21% and 22% of all deaths, respectively (4). Furthermore, every sixth male and every seventh woman in Europe will die from myocardial infarction (5).

Several factors have been investigated as potential contributors to the in-hospital death rate for ACS. A retrospective analysis conducted at a single tertiary cardiac center in Northeast Thailand identified risk variables for in-hospital mortality, that involves age >60 years, left ventricular ejection fraction <40%, and final TIMI flow grade 0/1 (6). In GRACE, eight risk factors accounted for 89.9% of the prognostic information including older age, higher Killip class, systolic blood pressure, ST-segment deviations, cardiac arrest during presentation, elevated serum creatinine level, positive initial cardiac enzyme finding, and heart rate (7).

A study from the ACCESS registry of South Africa identified clinical variables related with a greater risk of death at 12 months post-acute myocardial infarction, including age ≥70 years, diabetes mellitus on admission, and a history of stroke/transient ischemic attack (TIA) (8), while timely treatment with aspirin, clopidogrel, ACEIs, statins, and PCI was strongly related with low in-hospital mortality (9). According to the World Health Organization, CVD will be the main cause of mortality and morbidity by the end of 2030, with developing nations contributing significantly to this growth (10). For a long time, Africa, notably Ethiopia, suffered from communicable diseases and recently non-communicable diseases including cardiovascular diseases. Furthermore, ACS has recently become increasingly widespread in Ethiopia, with a poor prognosis (11).

A cross-sectional study on the spectrum of cardiovascular diseases at six major referral hospitals in Ethiopia in 2017 showed 6275 CVD patients, with IHD accounting for 9.6% of the cases. Only 334 (5.3%) of the patients came from SPHMMC (12). Although there is no formal registry for ACS in the country, retrospective studies conducted in 2019 on the clinical profile and outcome of ACS admitted over a two-year period at Tikur Anbessa Specialized Hospital on 142 cases revealed that male predominate with 99 (69.7%) of patients with a mean age of 57.8 years while females account the remaining part with a mean age of 56.9 years. Furthermore, the investigation found that the most common risk factors were a history of hypertension found in 101 (71.2%) patients, diabetes mellitus in 51 (35.9%), a history of myocardial infarction in 47 (33.1%), dyslipidemia in 21.8%, and a history of smoking cigarettes found in 12.7% (11) of study participants. Despite the large number of patients with acute coronary syndrome in Ethiopia, data on clinical patterns and overall treatment outcomes are limited, and even expected to be worse due to poor facilities in the setup. This study is therefore focused primarily on assessing the outcomes of acute coronary syndrome and associated variables in the two tertiary hospitals in Ethiopia. Furthermore, it also compared emergency management to standard guidelines, with the intention that the findings of this study could be utilized to implement hospital-based treatment standards, provide preventive health education, and plan community outreach activities targeting these segments of patients with potential risk of developing acute coronary syndrome.

## Methods

### Study design and setting

This was a retrospective study carried out in two public hospitals from January 2020 to February 2024, namely the SPHMMC and SPH located in the Capital, Addis Ababa. Medical chart records of hospitalized patients with ACS were used to examine outcomes and associated factors. The two hospitals serve roughly 300,000 individuals every year with a catchment population of more than 5 million. Both facilities are well-known for providing PCI care, which began before the study period and is active currently. The expected yearly medical ward and ICU admission rate was roughly 1,500 and 480 patients, respectively.

### Eligibility criteria

All patients with final diagnosis of at least one of the the three types of acute coronary syndrome including STEMI, NSTEMI, or unstable angina (UA) were included while those with the same diagnosis but whose medical records could not be accessed, those transferred to another hospital (except transfers between the two hospitals where the study was conducted), patients discharged against medical advice, and ACS patients who were 18 years old or younger, were excluded.

### Operational definition

• Treatment outcome: Treatment outcome of patients with ACS is explained by any death during hospital stay.
• STEMI: The universal definition of myocardial infarction was used to define STEMI based on ECG criteria (7). ACS subset defined by symptoms of acute myocardial infarction in association with persistent ST elevation with the release of biomarkers of myocardial necrosis.
• NSTEMI: ACS subset defined by symptoms of acute myocardial infarction in association with ST depression with the release of biomarkers of myocardial necrosis.
• Unstable angina (UA): ACS subset defined by symptoms of acute myocardial ischemia but no elevation in troponin, with or without ECG indicative of ischemia.
• Prior angina: History of angina before the current admission with acute coronary syndrome.
• Angina: refers to chest pain or pressure, with radiation to and causing jaw pain, arm pain, or other equivalent discomforts that is suggestive of cardiac ischemia.
• Previous MI: The patient has had at least 1 documented previous myocardial infarction before the current admission with acute coronary syndrome.
• Dyslipidaemia: The patient has had at least 1 documented dyslipidemia or who have elevated lipid profile level on current admission with acute coronary syndrome.
• Smoking History: confirming cigarette smoking in the past and or present:
  − Current: Smoking cigarettes within 1 month of this admission
  − Recent: Stopped smoking cigarettes between 1 month and 1 year before this admission
  − Former: Stopped smoking cigarettes greater than 1 year before this admission
  − Never: Never smoked a cigarette
• Killip class: Killip class of the patient at the time of current hospital admission:
  − Class I: Absence of rales over the lung fields and the S3 gallop
  − Class II: Rales over ≤50% of the lung fields or the presence of an S3
  − Class III: Rales over more than 50% of the lung fields
  − Class IV: Cardiogenic Shock
• Left Ventricular systolic dysfunction graded as:
  − Mild LVSD: Ejection fraction between 40%–49%
  − Moderate LVSD: Ejection fraction between 30%–39%
  − Severe LVSD: Ejection fraction of <30%

### Sampling procedures

Based on the computed sample sizes for the first and second objectives, an appropriate sample size to meet both objectives were 350 patients. With an incomplete and lost chart rate of 10%, the final sample size was 385. Admission and discharge registries from the Medical ICU and Medical Wards were utilized to determine the medical registration number of all patients admitted or discharged throughout the study period with the diagnosis of ACS. The identified MRNs were sorted by admission date and assigned numbers. Simple random sampling was utilized to select participant charts for each hospital using a computer-generated random number. Patients were stratified equally (1:1) from both hospitals. A total of 390 patient cards were retrieved; 82 of these were eliminated based on exclusion criteria, leaving a final total of 308 patient cards for data entry and analysis.

### Data collection

A checklist was developed from reviews of several standard pieces of literature and written in English, and the pretest was administered at Zewditu Memorial Hospital to 10% of the sample size and adjusted accordingly. Data was obtained from the patient's chart, which was chosen and identified according to inclusion criteria using a pretested and organized check list of queries. The primary investigator provided supervision to ensure completeness and consistency of the collected data.

### Data analysis and data quality control procedure

Data was cleaned, inputted, and analyzed with the Statistical Package for Social Science (SPSS version 27). Descriptive analysis was performed to describe the sociodemographic characteristics and the pattern of each independent variable. The significance of changes in these variables was tested using a chi-square test with a *P*-value of less than 0.05. Bivariable logistic regression analyses were used to evaluate the relationship between the dependent and independent variables and identify candidates for multivariable analysis. A multivariate analysis was then conducted on factors with *P*-value < 0.25 to identify the independent predictors of hospital mortality. All tests had a 95% confidence interval (CI) of *p* < 0.05, indicating statistical significance. The outcome was described by words, images, and tables. To ensure data quality, the following measures were implemented: an appropriately prepared data gathering checklist was used. The checklist or format was pre-tested. The lead investigator evaluated the collected data every day to ensure its completeness and consistency.

### Ethical consideration

SPHMMC and the SPH Ethical Review Committee both provided ethical clearance. An official letter was received from the department of internal medicine and delivered to the appropriate body at the medical ward and ICU. The information received from the records was kept confidential by substituting the patient's name with a unique code number.

## Results

### Sociodemographic characteristics of study participants

During the four-year period, a total of 308 patient charts with a diagnosis of ACS were evaluated, with an 88% retrieval rate. Of the total study participants, 223 (72.4%) were males, resulting in a male to female ratio of 2.6. Three-fourths of the subjects, 231 (75%) were from Addis Ababa City Administration, while 55 (17.9%) were from Oromia regional state. The average age was 56.3 (SD ± 13.5) years, with age range from 20 to 93 years. The majority of patients (39.0%) were between the ages of 50 and 64 years, with nearly one-third (31.2%) of study participants under the age of 50 years (Table 1).

**Table 1:** Sociodemographic characteristics of patients with acute coronary syndrome admitted to Saint Paul Hospital Millennium Medical College and Saint Peter Hospital, Addis Abeba, Ethiopia (2020–2024).

Variable	Category	*N* (%)
Age category	<50	96 (31.2%)
	50–64	120 (39.0%)
	≥65	92 (29.9%)
Sex	Female	85 (27.6%)
	Male	223 (72.4%)
Residence (Region)	Addis Ababa	231 (75.0%)
	Oromia	55 (17.9%)
	Amhara	8 (2.6%)
	Other	14 (4.5%)

### Presenting symptoms, time to presentation and risk factors among patients admitted with acute coronary syndrome

**Figure 1:**
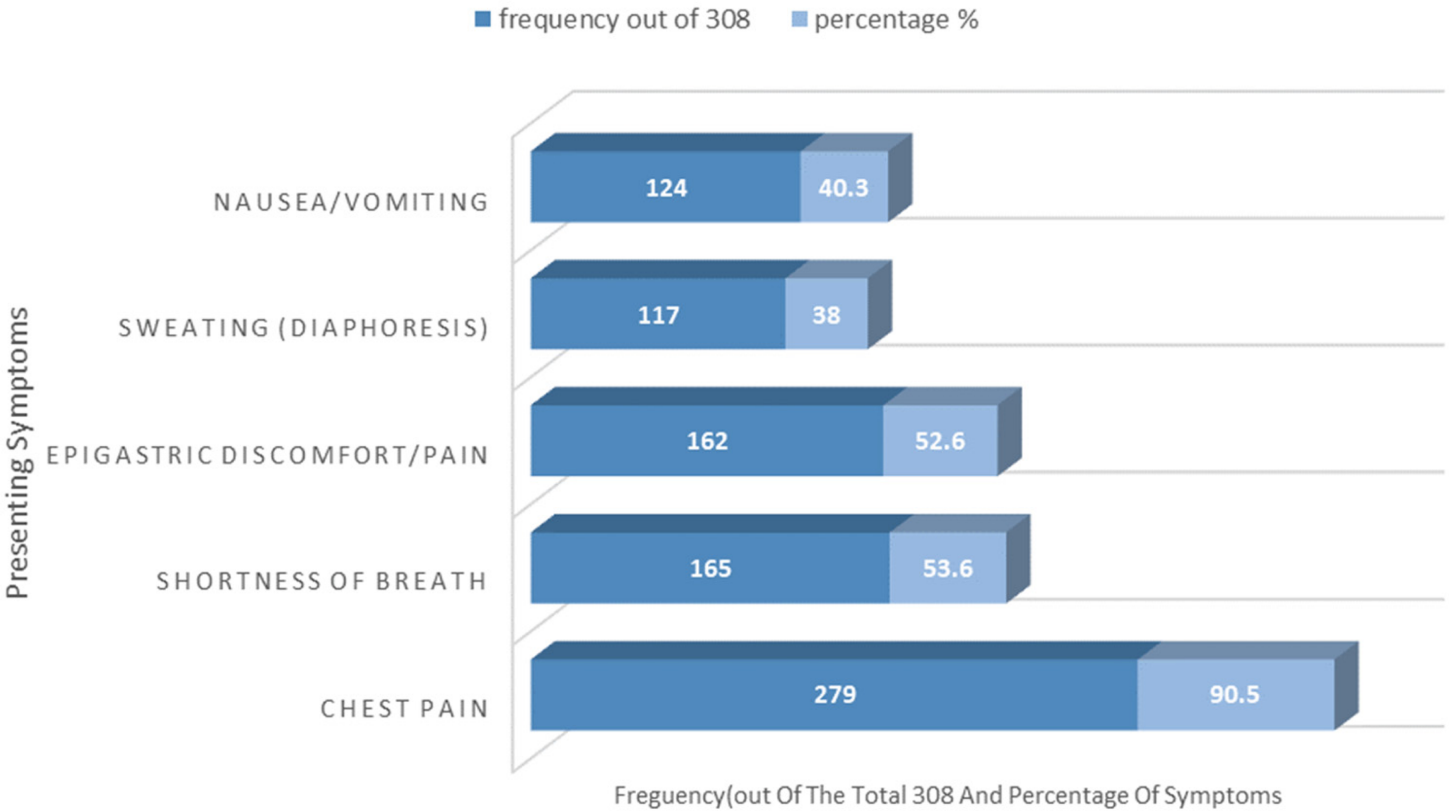
Presenting symptoms of patients with acute coronary syndrome admitted to Saint Paul Hospital Millennium Medical College and Saint Peter Hospital, Addis Abeba, Ethiopia (2020-2024).

The most commonly reported symptoms at presentation were chest pain (90.5%), followed by shortness of breath (53.6%) and epigastric discomfort/pain (52.6%). In terms of risk factors, hypertension was the most frequently detected (55.2%), followed by diabetes Mellitus (35.4%). About 27.3% had a previous history of angina, whereas 10.1% had a history of heart failure. Previous MI was noted in 8.1% of patients, and 8.8% had previous PCI. Smoking, CKD, dyslipidemia, and retroviral infection were all detected in approximately 5% of cases (Figure 1 and Table 2).

**Table 2:** Risk factors of patients with acute coronary syndrome admitted to Saint Paul Hospital Millennium Medical College and Saint Peter Hospital, Addis Abeba, Ethiopia (20202024).

Variable	Category	*N* (%)
Previous myocardial infarction	No	283 (91.9%)
	Yes	25 (8.1%)
Previous PCI	No	281 (91.2%)
	Yes	27 (8.8%)
Previous CABG	No	303 (98.4%)
	Yes	5 (1.6%)
Angina pectoris	No	224 (72.4%)
	Yes	84 (27.3%)
Previous stroke or TIA	No	303 (98.4%)
	Yes	5 (1.6%)
Diabetes	No	199 (64.6%)
	Yes	109 (35.4%)
Heart Failure	No	277 (89.9%)
	Yes	31 (10.1%)
Hypertension	No	138 (44.8%)
	Yes	170 (55.2%)
Family history of premature coronary heart disease	No	215 (69.8%)
	Unknown	86 (27.9%)
	Yes	7 (2.3%)
CKD	No	285 (92.5%)
	Unknown	6 (1.9%)
	Yes	17 (5.5%)
Dyslipidemia	No	292 (94.8%)
	Yes	16 (5.2%)
Smoking history	Current smoker	9 (2.9%)
	Former smoker	8 (2.6%)
	Never smoked	214 (69.5%)
	Unknown	77 (25.0%)
HIV infection	No	295 (95.8%)
	Yes, not on ART	4 (1.3%)
	Yes, on ART	9 (2.9%)

HIV, human immunodeficiency VIRUS; CKD, chronic kidney disease; PCI, percutaneous coronary intervention; CABG, coronary artery bypass grafting, Transient Ischemic Attack.

**Figure 2:**
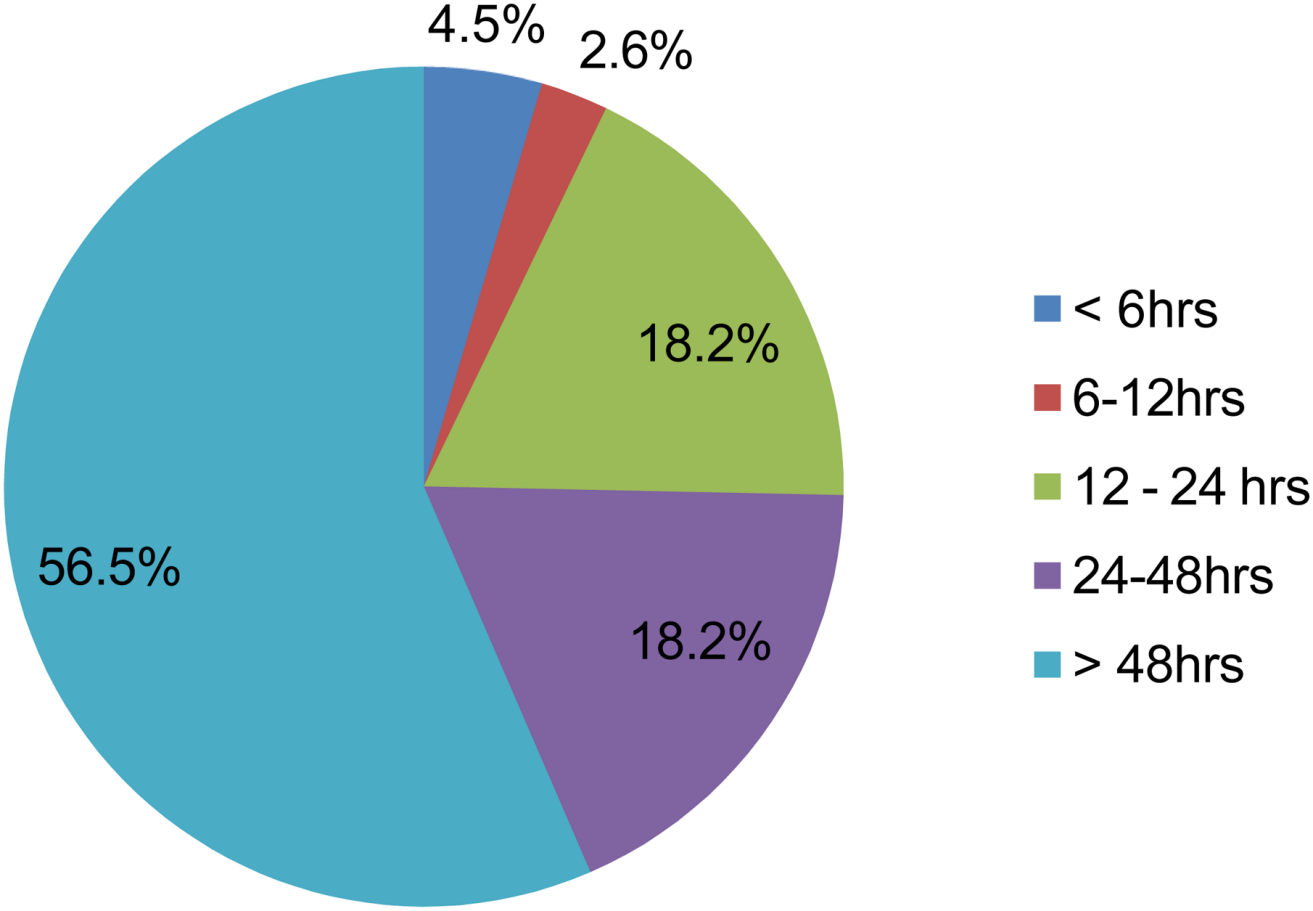
Time of arrival from symptom-onset of patients with acute coronary syndrome admitted to Saint Paul Hospital Millennium Medical College and Saint Peter Hospital, Addis Abeba, Ethiopia (2020-2024).

The mean presentation time to the Emergency Department (ED) after symptom onset was 3.7 days (SD ± 3.2), with a range of 2 h to 336 h (14 days). Fourteen participants (4.5%) reported to the emergency department within 6 h of symptom start, while 2.6% presented within 6–12 h, 18.2% between 12 and 24 h, and 18.2% within 24–48 h. The majority (56.5%) presented 48 h following symptom onset (Figure 2).

### Initial evaluation and killip class of study participants on presentation

**Figure 3:**
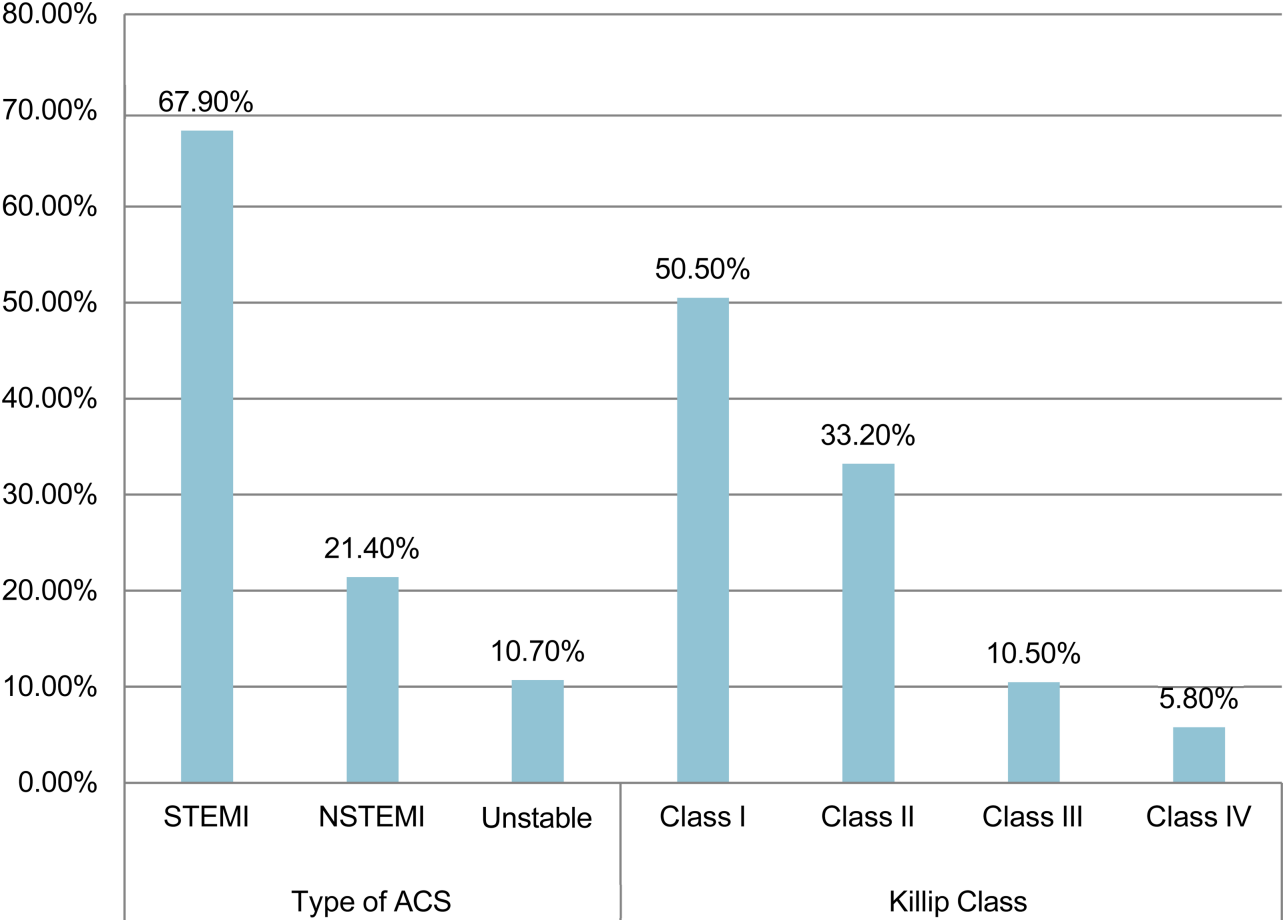
Types and killip classification of patients with acute coronary syndrome admitted to Saint Paul Hospital Millennium Medical College and Saint Peter Hospital, Addis Abeba, Ethiopia (2020-2024).

The mean systolic and diastolic blood pressure at presentation were 121.41 (SD ± 22.9) and 75.1 (SD ± 14.7), respectively. Approximately one-fifth of patients (18.9%) had systolic hypertension (SBP>=140/90 mmHG), while 7.1% had systolic hypotension (SBP <90 mmHG). At admission, the mean heart rate was 88.5 (SD ± 17.5), with 48.4% experiencing tachycardia (HR > 100), while approximately 6.2% of patients had ventricular tachycardia. Laboratory tests included serum troponin for 88.3% and CK-MB for 7.1% of patients, of which it was high in 89.3% of patients. The lipid profile was examined in 163 (52.9%) individuals, with mean total cholesterol, LDL, HDL, and TG (triglyceride) values of 172 mg/dl, 86 mg/dl, 55 mg/dl, and 118 mg/dl, respectively. STEMI accounted for nearly two-thirds (67.9%) of ACS cases, with NSTEMI and UA accounting for 21.4% and 10.7%, respectively. Of the 275 patients with indications of Killip class, 50.5% had Killip class I while 5.8% had Killip class IV at presentation (Figure 3).

On evaluation with echocardiography, 68.8% exhibited regional wall motion abnormalities, while 8.4% had LV thrombus, and yet 2.9% had an LV aneurysm. 42.9% of patients had reduced left ventricular ejection fraction (LVEF), with 8.4% having significant LV systolic dysfunction (Table 3).

**Table 3:** Echocardiographic findings of patients with acute coronary syndrome admitted to Saint Paul Hospital Millennium Medical College and Saint Peter Hospital, Addis Abeba, Ethiopia (2020–2024).

Variable	Category	*N* (%)
LVEF	≥50%	176 (57.1%)
	40–49%	47 (15.3%)
	30–39%	59 (19.2%)
	<30%	26 (8.4%)
Regional wall motion abnormality	No	96 (32.8%)
	Yes	212 (68.8%)
LV thrombus	No	282 (91.6%)
	Yes	26 (8.4%)
LV Aneurysm	No	252 (97.1%)
	Yes	9 (2.9%)
Mitral Regurgitation	No	172 (55.8%)
	Mild	100 (32.5%)
	Moderate	30 (9.7%)
	Severe	6 (1.9%)
RV Infarction	No	303 (98.4%)
	Yes	5 (1.6%)
Pericardial Effusion	No	281 (91.2%)
	Mild	16 (5.2%)
	Moderate	7 (2.3%)
	Present but Not graded	4 (1.5%)

### In-hospital management and treatment outcome of patients with acute coronary syndrome

Almost all patients (97.7%) received aspirin, while 93.3% had clopidogrel, and 91.2% received anticoagulant medication as part of initial medical treatment. Statins and beta blockers were administered to 91.6% and 59.8% of patients, respectively. ACEis/ARBS was administered to 75.3% of the patients. None of the STEMI patients that came within the therapeutic window (*n* = 8; 3.8%) got thrombolytic treatment. This was due to various factors, including contraindications, patient refusal, patient preference for PCI, and undocumented reasons. In the meantime, further investigations were done for some of the patients presented with acute coronary syndrome. Fifty-four (17.5%) of the patients underwent coronary angiography during their index hospitalization, of whom thirty-eight (12.3%) of them underwent percutaneous coronary intervention (PCI), limited to those who met the standard international guideline recommendations. Follow-up angiography was performed in 13.3% of cases based on evidence of disease progression, recurrent symptoms, or complications, including reinfarction. Among these, PCI was conducted in 5.2% of the cases. The average time for angiography and PCI was 7.4 (SD ± 13.4) and 8.5 (SD ± 14.5) days, respectively (Table 4).

**Table 4:** In-hospital cocktail of management profile of patients with acute coronary syndrome admitted to Saint Paul Hospital Millennium Medical College and Saint Peter Hospital, Addis Abeba, Ethiopia (2020–2024).

Drugs given		*N* (%)
Aspirin		301 (97.7%)
Clopidogrel		288 (93.5%)
Beta blockers		215 (59.8%)
ACEi/ARBs		232 (75.3%)
Statins		282 (91.6%)
Morphine		136 (44.2%)
Nitrates		20 (6.5%)
Anticoagulants	Unfractionated heparin	281 (91.3%)
	Enoxaparin	5 (1.6%)
Coronary Angiography done	During index hospitalization	54 (17.5%)
	On subsequent follow-up	41 (13.3%)
PCI done	Yes, during index hospitalization	38 (12.3%)
	Yes, during subsequent follow-up	16 (5.2%)

**Figure 4:**
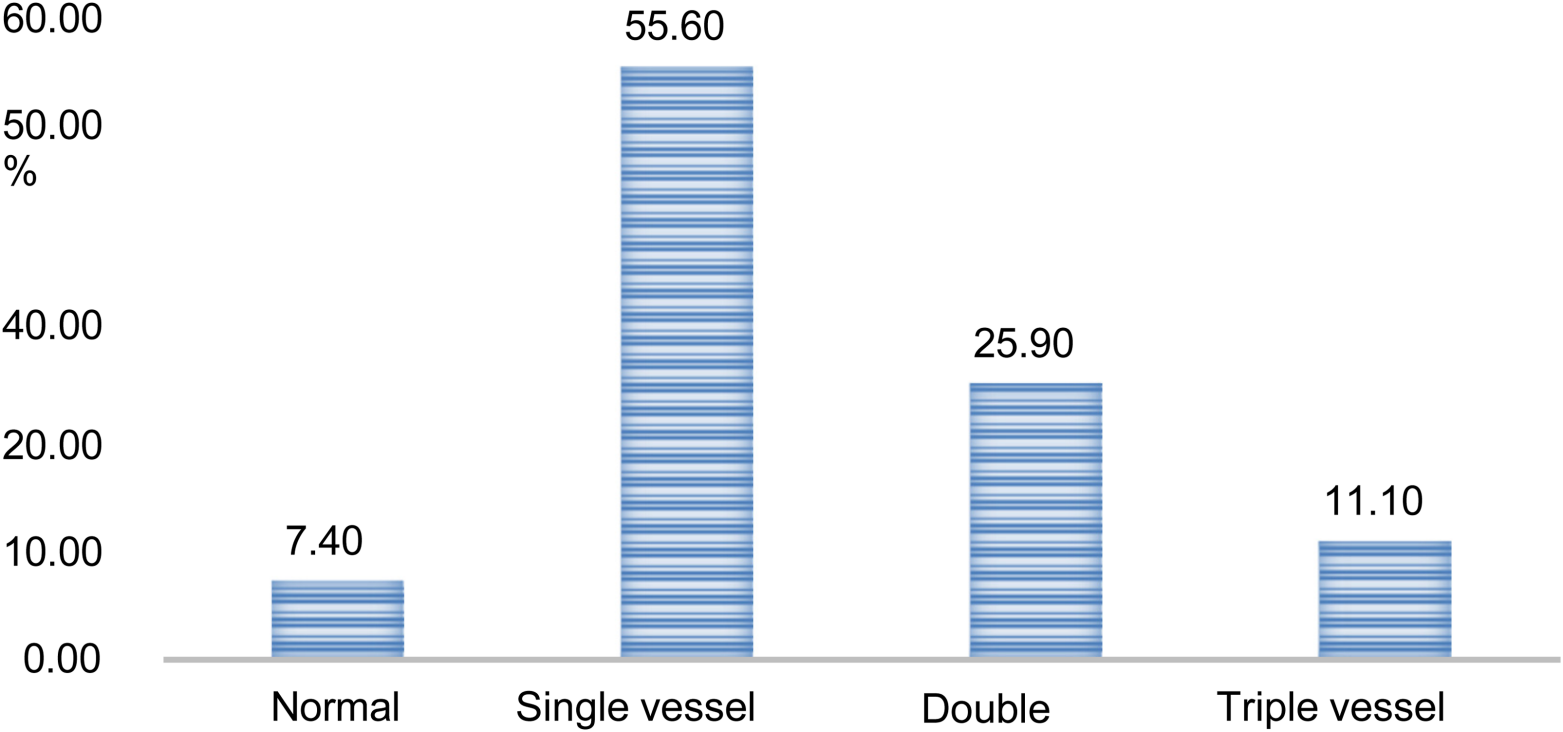
Outcomes of coronary angiography of patients with acute coronary syndrome admitted to Saint Paul Hospital Millennium Medical College and Saint Peter Hospital, Addis Abeba, Ethiopia (2020-2024).

From the total number of coronary angiographies performed (*n* = 95, 30.8%), 7.4% were found to be normal, while more than half of patients (55.6%) had single vessel disease while 37% had double or triple vascular disease. LAD is the most often diseased vessel (in isolation or with other arteries), accounting for 62% of all cases (Figure 4).

### Outcomes and predictor of in-hospital mortality of patients with acute coronary syndrome

**Figure 5:**
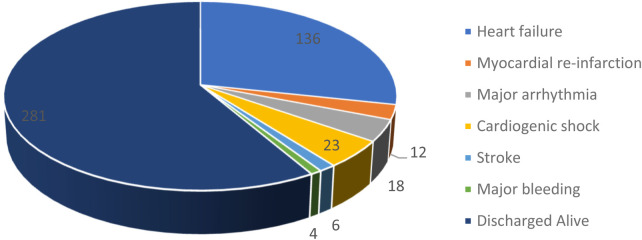
Outcomes of patients with acute coronary syndrome admitted to Saint Paul Hospital Millennium Medical College and Saint Peter Hospital, Addis Abeba, Ethiopia (2020-2024).

In terms of in-hospital outcomes, heart failure was the most frequent ACS-related complication, occurring in approximately 44.1% of patients, followed by cardiogenic shock (7.5%). About 5.8% of patients developed major arrhythmias, while 3.9% of the patients experienced myocardial re-infarction. In terms of treatment success, 91.2% of patients were discharged with improvement, while in-hospital mortality was 8.8%, of whom 81.5% were those diagnosed with STEMI, 11.1% with NSTEMI, and 7.4% with UA. Within the various ACS groups, 22 (10.5%) of patients with STEMI, 3 (4.5%) of patients with NSTEMI, and 2 (6.2%) in the UA group died. The average length of hospital stays (in days) before the final outcome was 8.51 (SD = 7.2) (Figure 5).

Several variables were shown to be substantially linked with treatment outcome on crude analysis, including pulse rate, systolic blood pressure, serum creatinine, LVEF, beta blocker use, and Killip class. Multivariable logistic regression analysis revealed that all variables, except systolic blood pressure and serum creatine, were substantially linked with treatment outcome (*p*-value < 0.05). As a result, pulse rate above 140 increased the risks of mortality by 7.5 times (AOR = 7.50, 95% CI: 1.36, 41.57) when compared to the reference group. Patients in Killip class IV had a 6.03 times higher risk of mortality than those in Killip class I (AOR = 6.03, 95% CI: 1.27, 28.61). Similarly, individuals with slightly, moderately, and significantly decreased LVEF had 5.12 times (AOR = 5.12 95% CI: 1.13, 23.12),5.29 times (AOR = 5.29 95% CI: 1.26, 22.29) and 7.46 times (AOR = 7.4695% CI: 1.40, 39.65) increased risk of mortality respectively compared to those with normal EF. Beta blockers were associated with an 83% lower risk of mortality when compared to individuals who did not take the medicine (AOR = 0.17, 95% CI: 0.05, 0.63) (Table 5).

**Table 5:** Associated factors of in-hospital mortality among patients with acute coronary syndrome admitted to Saint Paul Hospital Millennium Medical College and Saint Peter Hospital, Addis Abeba, Ethiopia (2020–2024).

Variable	Alive (*N*)	Died (*N*)	COR	AOR	95% CI for AOR		*P*-value
					Lower	Upper	
PR ≥141	62	13	5.032*	7.515	1.358	41.574	.021
Cr >1.2	43	10	3.33**	2.715	.087	8.525	.204
Killip Class IV	8	8	12.900*	6.028	1.270	28.605	.024
SBP <90	16	6	3.616*	3.359	.725	15.552	.121
LVEF 40–49	41	4	3.455*	5.116	1.133	23.106	.034
LVEF 30–39	49	9	5.782**	5.292	1.256	22.286	.023
LVEF <30	21	5	8.095**	7.462	1.404	39.652	.018
Beta blocker use	201	14	0.42*	.171	.046	.631	.008

*P*-value <0.05 *>0.01; **</=0.01.

PR, pulse rate; LVEF, left ventricular ejection fraction; SBP, systolic blood pressure; Cr, serum creatinine; AOR, adjusted odd ratio; COR, crude odd ratio.

## Discussion

The age distribution of ACS patients in this study was 56.3 ± 13.5 years, which is in line with the studies done at Tikur Anbessa Specialized Hospital (56.3 ± 13.6 years), Ayder Hospital (59.12 ± 12.98 years), Kenya (59.7 ± 3.8 years), and India (60.1 ± 11.21 years), but lower than that of the Global Registry of Acute Coronary Events (The GRACE registry) (64.9 ± 12.6 years) (2, 6, 11, 13–15). The study found a substantial delay in seeking medical care (mean time from symptom onset was 3.7 days (SD ± 3.2), as evidenced from the fact that only 4.5% of participants presented to the ED within 6 h and 7.1% within 12 h after symptom onset, with the majority (56.6%) presenting after 48 h. Local studies conducted at Ayder Hospital [average time to presentation 4 days (SD ± 6.0)] (13) and Tikur Anbessa Hospital (mean time of presentation to ED 3.8 days) (11) support similar time delay.

According to research conducted in Kenya, 78% of patients came within 12 h after symptom onset (16), while another research conducted in Dakar; Senegal found that the average time delay before receiving medical attention was 14.5 h (17). The ENACT study, a pan-European review of acute coronary syndromes, found that the majority of patients (65%) presented within 12 h of the onset of discomfort (18). The significant delay in our study is primarily due to limited access to PCI centers and referrals to a few public treatment facilities. Additional factors include geographic barriers, financial constraints, overcrowded centers, and low public awareness about myocardial infarction symptoms. Cultural beliefs, reliance on traditional healers, and underdeveloped emergency medical services further exacerbate delays.

STEMI is the most common type of ACS, accounting for nearly two-thirds (67.9%), whereas NSTEMI and UA accounted for 21.4% and 10.7% respectively. The proportion of STEMI is consistent with local studies conducted at Tikur Anbessa Hospital (72.6%) (11) and Ayder Hospital (67.5%) (13), but greater than the GRACE trial (49.5%), SPACE Registry (41.5%), and ACCESS Registry of South Africa (46%) (5, 19). The higher proportion of STEMI cases in our study may be linked to delays in seeking medical attention. NSTEMI and UA, caused by partial occlusion, can progress to STEMI due to complete blockage. Killip class I made up over half of the cases (50.5%), followed by Killip class II (33.2%) while Killip class III and IV accounted for 16.3% of cases (10.5% and 5.8%, respectively), which is consistent with the GRACE registry (15.3%) though lower than the studies conducted at Tikur Anbessa (54.2%) and Ayder Hospital (43.7%) (11, 13, 20). Several factors may explain including differences in patient population, with potentially fewer comorbidities or better baseline health, as well as variations in treatment protocols such as early PCI or effective medical management. Additionally, the level of healthcare infrastructure and medical expertise at the study facility, along with potentially quicker medical intervention and more effective monitoring, may have contributed to better outcomes and fewer complications.

In terms of hospital management, aspirin was loaded in 97.7% (similar to studies conducted in South Africa (94%), Kenya (98%), Italy (92.8%), and the GRACE registry (92%), and higher than local studies conducted at Tikur Anbessa Hospital (79%) and Ayder Hospital (63.8%) (5, 11, 13, 20, 21). Clopidogrel was likewise loaded at 93.3%, while the use of DAPT (Dual antiplatelet treatment) in this study was consistent with the guidelines' recommendations. The increased use of DAPT in this study could be attributed to the large number of patients presenting with chest discomfort (90.5%) and improved documentation.

Beta blockers were administered to 59.8% of patients, which is lower than in local studies conducted at Tikur Anbessa (88%), Ayder Hospital (86%), the GRACE Registry (76%), and South Africa (69%) (5, 11, 13, 16). None of the patients in this study received thrombolytic therapy, despite the fact that 4.5% of patients presented to the ED within 6 h of symptom onset, with probable explanations including local trend, unavailability and cost difficulties. Other studies conducted at TASH (0%), Ayder Comprehensive Specialized Hospital (3.9%), Kenya (5%), and South Africa (26%), found similar rates of thrombolytic use (11, 13, 21, 22).

Percutaneous coronary intervention (PCI) was performed on 38 (12.3%) of patients during the same index hospitalization, which is comparable to studies conducted in Kenya (12%), and South Africa (19%), but higher than studies conducted at Ayder Comprehensive Specialized Hospital (3.9%) (13, 21, 22). Despite the fact that PCI services were initiated in the research environment, only a small number of patients benefited from them. Possible factors include late presentation, a lack of a normally functional cath lab (which is only available two days a week in this context), and interruption of supplies. Heart failure was the most common in-hospital complications (44.1%), followed by cardiogenic shock that was found in 7.5%, severe arrhythmia (5.8%), and myocardial re-infraction (3.9%). According to a study conducted in Egypt, the most common ACS complications was heart failure (33.6%), followed by ventricular tachycardia (12.5%) and cardiogenic shock (10.5%), nearly similar to this study. The increased rate of heart failure in this study could be attributed to late presentation and a lack of reperfusion treatment.

This study found that 8.8% of patients died in the hospital. The majority of deaths (81.5%) were from the STEMI sub-group, which can be explained by the higher number of STEMI cases (67.9%), while 11.1% and 7.4% came from NSTEMI and UA, respectively. This finding is lower than that of Tikur Anbessa (27.4%), Ayder hospitals (24.5%), Nigeria (21.4%), Senegal (21%), India (18.4%), and Kenya (17%), and yet comparable to other research from Pakistan (12.2%), Kenya (9.4%), and Sub-Saharan African nations (10%) (9, 11, 13, 22, 23). This study also demonstrated virtually similar mortality with other large-scale studies and registries; stating in-hospital mortality of 7.66% in the Meta-analysis of 2,128 patients from 20 tertiary hospitals in Heilongjiang Province, China done in 2007 (19), and 9.6% in a study done at 32 hospitals.

The mortality rates for STEMI, NSTEMI, and UA were 10.5, 4.5, and 6.1%, respectively, in this study. In 2003, the GRACE registry reported an in-hospital death rate of 7% for STEMI, 4% for NSTEMI, and 3% for UA patients, which is also consistent with this study (6). The comparatively low fatality rate in this study could be attributed to a lower proportion (16.3%) of patients with higher Killip class (Killip class III and IV) as compared to other local studies conducted at Tikur Anbessa (54.2%) and Ayder Hospital (43.7%) (11, 13). There were no confirmed deaths in the PCI group, however this could be related to selection bias because PCI was usually done for stable patients and not during the acute phase of the illness.

Multivariate logistic regressions were used to remove confounding factors and found that decreased LVEF (EF < 50%), tachycardia (PR > 140), Killip class IV, and non-initiation of beta blockers were significantly associated with in-hospital mortality in patients with ACS in this study. In GRACE, age, Killip class, systolic blood pressure, ST segment deviation, cardiac arrest during presentation, serum creatinine level, positive initial cardiac enzyme result, and heart rate were all predictive of in-hospital mortality (20). This study found that starting Beta blockers reduced the risk of death by 83% compared to not taking the medicine (AOR = 0.17, 95% CI: 0.05, 0.63). Similarly, several trials conducted at Ayder Hospital in Brazil, Switzerland, and Russia found that early start of beta blockers reduced in-hospital mortality (13, 24–26).

LVEF at admission is a significant predictor of death in ACS as well (27). Furthermore, the study found that LV Systolic dysfunction (LVEF <50%) increases the risk of mortality by 5.12 times (AOR = 5.12 95% CI: 1.13, 23.12), 5.29 times (AOR = 5.29 95% CI: 1.26, 22.29), and 7.46 times (AOR = 7.4695% CI: 1.40, 39.65) for mildly, moderately, and severely reduced LVEF, respectively. This is consistent with ACSIS surveys conducted in Israel on a total of 11,536 patients between 2000 and 2010, which showed severe LV dysfunction (LVEF <30%) and mild to moderate LV dysfunction (LVEF 30–49) had 4.49- and 1.83-fold higher mortality risk compared to the LVEF >50% group serving as the reference group (HR 4.49; [95% CI3.57–5.61 and HR 1.83; (1.49–2.24), respectively] (27, 28).

### Strength and limitations

The sample size in the study was adequate and participants were chosen using a basic random sampling procedure, which minimizes selection bias. However, it has drawbacks. As it was a two-center study, it could not represent the entire hospital population, particularly privately held institutions having access to revascularization therapy. The study's retrospective nature has also had an impact on the completeness of the required information. Some variables had a high number of missing values, which could have caused bias with a broad confidence interval. As a result, the study's overall findings may not be generalizable to the general population.

## Conclusion

The study discovered a significant number of STEMI patients who were treated medically, owing to delayed presentation, restricted availability of PCI facilities, and inconsistent supply of consumables in the setup. Though dual antiplatelet therapy was optimum, the usage of beta blockers was significantly low, despite the lack of contraindications. A significant proportion of patients developed heart failure and cardiogenic shock, culminating in in-hospital mortality. Health education on the signs and symptoms of acute myocardial infarction, together with increased service availability, would reduce complications and improve survival rates among patients with acute coronary syndrome in the setting.

## Data Availability

The original contributions presented in the study are included in the article/Supplementary Material, further inquiries can be directed to the corresponding author.
